# From Early Diet to Immune Phenotype: Childhood Nutrition and Immunometabolic Programming Across the Life Course—A Scoping Review

**DOI:** 10.3390/nu18213706

**Published:** 2026-11-08

**Authors:** Lucian-Ioan Cristun, Otilia Mărginean, Iulius Jugănaru, Raluca-Nicoleta Vasilescu, Niculina Mang, Andrei-Ioan Munteanu, Delia-Maria Nicoară, Meda Ada Bugi, Andrei Păun, Alexandra-Diana Cheleș, Laura Cristina Rusu

**Affiliations:** 1 Doctoral School, University of Medicine and Pharmacy, 300041 Timișoara, Romania; lucian.cristun@umft.ro (L.-I.C.); andrei.paun@umft.ro (A.P.); alexandra.cheles@umft.ro (A.-D.C.); 2 First Pediatric Clinic, Victor Babeș University of Medicine and Pharmacy, 300041 Timișoara, Romania; marginean.otilia@umft.ro (O.M.); nina.mang@umft.ro (N.M.); andrei-ioan.munteanu@umft.ro (A.-I.M.); nicoara.delia@umft.ro (D.-M.N.); 3 Research Center for Growth and Development Disorders in Children (CREDE), 300041 Timișoara, Romania; 4 Louis Țurcanu Emergency Clinical Hospital for Children, 300041 Timișoara, Romania; raluca.bolboase@gmail.com; 5 Department of Pharmacy, University of Medicine and Pharmacy ‘Vasile Goldis’, 310414 Arad, Romania; 6 Multidisciplinary Center of Research, Evaluation, Diagnosis and Therapies in Oral Medicine, Victor Babeș University of Medicine and Pharmacy, 300041 Timișoara, Romania; laura.rusu@umft.ro; 7 Clinic of Oral Pathology, Victor Babeș University of Medicine and Pharmacy, 300041 Timișoara, Romania

**Keywords:** childhood nutrition, immunometabolism, immune programming, gut microbiome, dietary diversity, allergic sensitization, developmental timing, life-course nutrition

## Abstract

Childhood diet may shape immune and inflammatory trajectories through interacting metabolic, microbial, and developmental pathways, but the long-term human evidence remains heterogeneous. This scoping review mapped longitudinal studies examining dietary exposures from birth to 18 years in relation to subsequent immune, inflammatory, allergic, autoimmune, gastrointestinal, immunometabolic, hepatic, neurocognitive, and endocrine–immunometabolic outcomes. The review was conducted in accordance with Joanna Briggs Institute guidance and reported according to PRISMA-ScR principles. A total of 403 studies were included. Evidence was concentrated in infancy and early childhood and predominantly addressed allergic and atopic outcomes; evidence on other immunological outcomes and on middle childhood and adolescence was more limited. Thirty studies provided relatively direct immunometabolic evidence, 149 contributed mechanistically indirect evidence, and 224 primarily reported longitudinal clinical diet–outcome associations. The principal mechanistic axes involved allergic sensitization and immune tolerance, lipid and fatty-acid metabolism, micronutrient and antioxidant pathways, cytokine signaling, gut microbiome and metabolite pathways, and human-milk-derived bioactive signals. Dietary diversity and overall diet quality intersected with several of these pathways. Associations were frequently context-dependent, varying according to developmental timing, host susceptibility, genetic background, immune state, and feeding context. Mechanistic interpretation rests on convergence across complementary studies rather than on complete pathways demonstrated within individual cohorts. Overall, the evidence is compatible with a proposed life-course nutritional immunology framework in which childhood diet acts as a dynamic developmental exposure interacting with microbial, metabolic, and immune processes; however, this framework derives mainly from early-life exposures and allergic and atopic outcomes, and the persistence of effects beyond early childhood remains insufficiently demonstrated. Limitations include restriction to English-language publications, marked methodological heterogeneity, residual confounding, and the absence of a formal risk-of-bias assessment.

## 1. Introduction

Childhood is a period of marked nutritional, metabolic, microbial, and immunological plasticity. From infancy through adolescence, dietary exposures provide not only the substrates required for growth and development but also biological signals capable of influencing immune maturation and regulation. This is particularly relevant during early life, when immune competence, intestinal barrier function, and the gut microbiome develop in parallel and remain highly responsive to environmental influences. Breastfeeding, formula feeding, complementary feeding practices, dietary diversity, nutrient composition, and later dietary patterns may therefore contribute to the establishment of immune phenotypes with consequences extending beyond childhood [1,2,3].

The relationship between nutrition and immunity extends well beyond the prevention of classical nutrient deficiencies. Dietary components may modulate innate and adaptive immune responses through interconnected mechanisms involving cytokine production, immune-cell differentiation, epithelial barrier integrity, oxidative balance, and metabolic signaling. Diet is also a major determinant of gut microbial composition and function, particularly during early development. Microbial metabolites, including short-chain fatty acids and other bioactive compounds, can interact with epithelial, metabolic, and immune pathways and contribute to immune maturation and tolerance [1,2,3]. Together, these interactions provide a biologically plausible basis for the concept of immune and inflammatory programming, whereby nutritional exposures during critical developmental windows may influence long-term immune and inflammatory regulation and subsequent susceptibility to allergic, autoimmune, inflammatory, and metabolic disorders [2,3].

This programming is unlikely to depend on a single nutrient or food. Rather, it may reflect the combined influence of dietary timing, quality, diversity, and inflammatory potential, together with microbiome development, intestinal barrier function, genetic susceptibility, adiposity, and other environmental exposures. Prospective pediatric evidence supports the relevance of this framework, with persistent unfavorable dietary patterns during childhood being associated with higher subsequent levels of systemic inflammatory biomarkers such as high-sensitivity C-reactive protein [4].

Importantly, the consequences of childhood dietary exposure may emerge only after a substantial time interval. Longitudinal studies suggest that nutritional characteristics during infancy, childhood, and adolescence may be associated with clinical or biological phenotypes later in life. Early-life diet quality and specific food exposures have been prospectively associated with subsequent inflammatory bowel disease, supporting a potential relationship between the early nutritional environment and later immune-mediated gastrointestinal disease [5]. Similarly, greater adherence to a Mediterranean-style dietary pattern during childhood and adolescence has been associated with a more favorable cardiometabolic profile in young adulthood [6]. These observations support the need to examine childhood nutrition within a life-course framework, rather than limiting assessment to contemporaneous pediatric outcomes.

The consequences of nutritional programming may extend across multiple organ systems linked by shared inflammatory and metabolic pathways. The gut–liver axis represents one potential route through which childhood diet, microbial activity, insulin resistance, and metabolic inflammation may influence hepatic fat accumulation and later metabolic liver disease. Prospective evidence has linked childhood energy intake with markers of non-alcoholic fatty liver disease during adolescence [7]. The microbiota–gut–brain axis may represent another interface connecting diet, microbial metabolites, metabolic signaling, immune regulation, and brain development. Longitudinal studies have reported associations between adolescent dietary patterns and subsequent cognitive performance [8], while dietary trajectories beginning in youth have also been examined in relation to cognitive function later in adulthood [9]. However, because inflammatory or immune pathways are not always directly measured in neurocognitive studies, such outcomes should be distinguished from evidence supported by direct immunological or inflammatory measures.

The developmental timing of dietary exposure is also likely to be important. Nutritional exposures during infancy occur alongside rapid microbiome development, intestinal maturation, and the establishment of immune tolerance [1,2,3], whereas later childhood and adolescence are characterized by major changes in body composition, endocrine regulation, metabolic demands, and dietary autonomy. Associations observed during one developmental stage may persist, attenuate, or change over time, as suggested by longitudinal evidence across gastrointestinal, inflammatory, metabolic, hepatic, and neurocognitive outcomes [4,5,6,7,8,9]. Therefore, both the developmental window of exposure and the age at outcome assessment are central to understanding long-term nutritional programming.

Despite increasing interest in these relationships, the available literature remains fragmented. Breastfeeding, individual nutrients, dietary patterns, microbiome development, systemic inflammation, and specific disease outcomes have often been investigated separately [1,2,3,4]. Considerable heterogeneity also exists in exposure definitions, developmental periods, biomarkers, clinical outcomes, follow-up durations, and study designs. As a result, the broader relationship between childhood dietary exposures and subsequent immune and inflammatory trajectories across organ systems and into adulthood remains incompletely characterized.

A scoping review is therefore well suited to map this heterogeneous evidence base. Rather than focusing on a single dietary intervention or estimating a pooled effect, the present review seeks to characterize how childhood nutrition has been investigated in relation to subsequent immune and inflammatory health, identify the developmental windows and biological pathways most frequently studied, distinguish direct immune and inflammatory evidence from mechanistically indirect life-course associations, and identify areas in which longitudinal evidence remains limited.

Accordingly, this scoping review aims to map and characterize the available evidence on long-term immune, inflammatory, allergic, autoimmune, gastrointestinal, immunometabolic, hepatic, neurocognitive, and endocrine–immunometabolic outcomes associated with dietary exposures occurring during childhood, with particular emphasis on developmental timing, persistence of effects, and potential mechanisms of immune programming.

The primary research question is: What evidence exists regarding long-term immune, inflammatory, allergic, autoimmune, gastrointestinal, immunometabolic, hepatic, neurocognitive, or endocrine–immunometabolic outcomes associated with dietary exposures occurring during childhood? A secondary objective is to determine whether the type and timing of childhood dietary exposure influence the direction, magnitude, or persistence of subsequent immune and inflammatory outcomes across the life course.

## 2. Materials and Methods

### 2.1. Study Design and Reporting Framework

This scoping review was conducted in accordance with the methodological guidance of the Joanna Briggs Institute (JBI) for scoping reviews [10] and reported according to the Preferred Reporting Items for Systematic Reviews and Meta-Analyses Extension for Scoping Reviews (PRISMA-ScR) [11].

The review was designed to map the extent, characteristics, and direction of the available evidence linking dietary exposures occurring during childhood with subsequent immunological, inflammatory, metabolic, and related life-course outcomes.

The review protocol was not prospectively registered in a public registry.

Reporting was guided by the PRISMA-ScR checklist [11], and the study-selection process is presented in a PRISMA-ScR flow diagram (Figure 1).

### 2.2. Review Question and Eligibility Framework

Eligibility criteria were structured according to the Population–Concept–Context (PCC) framework.

The primary review question was:

What evidence exists regarding long-term immune, inflammatory, allergic, autoimmune, gastrointestinal, immunometabolic, hepatic, neurocognitive, or endocrine–immunometabolic outcomes associated with dietary exposures occurring during childhood?

The population of interest comprised individuals whose relevant dietary exposure occurred between birth and 18 years of age. Eligibility was defined according to age at exposure rather than age at outcome assessment; therefore, outcomes assessed during later childhood, adolescence, young adulthood, or adulthood were considered eligible.

The concept of interest was childhood dietary exposure in relation to subsequent immune programming and immune and inflammatory trajectories. Eligible exposures included breastfeeding, formula feeding, complementary feeding, timing of food introduction, individual foods and food groups, macro- and micronutrients, dietary patterns, diet quality and diversity, Mediterranean- and Western-type dietary patterns, ultra-processed foods, dietary inflammatory potential, and other clearly defined nutritional exposures occurring during childhood.

Studies assessing maternal prenatal diet alone were excluded unless a distinct postnatal dietary exposure of the child was also reported.

Eligible outcomes included immune and inflammatory biomarkers; allergic and atopic outcomes; autoimmune and immune-mediated diseases; gastrointestinal and gut-mediated outcomes; immunometabolic phenotypes; hepatic and metabolic liver outcomes; neurocognitive outcomes; and endocrine–immunometabolic outcomes. At the full-text stage, immune or inflammatory biomarkers were considered eligible when they were assessed in relation to a subsequent clinical immune-mediated outcome; studies reporting biomarkers only, without a clinical outcome, were not included.

Neurocognitive outcomes without direct measurement of immune or inflammatory pathways were retained as mechanistically indirect secondary outcomes and were considered separately from evidence based on direct immunological or inflammatory measures.

### 2.3. Temporal Eligibility and Definition of Subsequent Outcomes

A predefined temporal relationship between dietary exposure and outcome was required. The relevant dietary exposure had to occur between birth and 18 years of age and precede the subsequent outcome assessment.

For the purposes of this review, subsequent outcomes were defined as eligible outcomes assessed at a later developmental stage or follow-up time point after the childhood dietary exposure. The term “long-term” is used descriptively and does not, by itself, imply persistence of an effect.

No minimum follow-up duration was imposed, for two reasons. First, the aim of a scoping review is to map the extent and nature of the available evidence, including its gaps, rather than to estimate the effect of exposures with a given latency. Second, the time required for an outcome to become manifest differs markedly between the eligible outcomes: eczema, food allergy, and allergic sensitization typically manifest in infancy and early childhood, whereas hepatic, metabolic, and neurocognitive outcomes generally require follow-up into adolescence or adulthood. A single threshold would therefore have excluded much of the early-life allergic literature or applied an inappropriate standard to outcomes with longer latency, without improving the assessment of persistence.

To avoid ambiguity, follow-up duration—defined as the interval between the dietary exposure and the latest outcome assessment—was charted for every included study and categorized as <5 years, 5–10 years, >10 years, or outcome assessed in adulthood. A predefined interpretive rule was applied: associations with outcomes assessed <5 years after exposure were considered short-term and were not interpreted as evidence of persistent biological programming; a follow-up of 5–10 years was considered intermediate; and only outcomes assessed more than 10 years after exposure or in adulthood were considered relevant to persistence, and even these were interpreted as long-term associations rather than as direct evidence of persistent biological programming.

Studies in which dietary exposure and outcome were assessed only concurrently, without an identifiable temporal relationship, were excluded.

### 2.4. Types of Evidence Sources

Eligible evidence sources comprised primary human studies, including prospective and retrospective cohort studies, birth cohorts, case–control studies with a clearly established temporal relationship between exposure and outcome, randomized or non-randomized dietary intervention studies with longitudinal follow-up, and other longitudinal observational designs.

Purely cross-sectional studies, case reports, small case series, editorials, commentaries, and letters were excluded. Systematic reviews and meta-analyses were not included as primary evidence but were used, where relevant, for background information and citation tracking. Animal and in vitro studies were excluded from the main analysis.

Studies evaluating dietary interventions initiated after diagnosis solely for the treatment or control of an established inflammatory, autoimmune, gastrointestinal, metabolic, hepatic, or endocrine disorder were excluded unless they also provided independent evidence relevant to subsequent long-term immune programming.

Only English-language studies published between 1 January 2000 and 31 December 2025 were considered eligible.

### 2.5. Information Sources and Search Strategy

The electronic literature search included MEDLINE via PubMed, Scopus, and Web of Science Core Collection.

The final searches in all three databases were conducted on 12 September 2026, with the date of the last search for each database reported in Table S1.

The search strategy was developed around three main conceptual domains:

1. Childhood or early-life exposure;
2. Nutrition or dietary exposure;
3. Subsequent immune, inflammatory, allergic, autoimmune, gastrointestinal, immunometabolic, hepatic, neurocognitive, or endocrine–immunometabolic outcomes.

For PubMed, controlled vocabulary terms were combined with free-text Title/Abstract terms. The search strategies were adapted to the indexing structure and syntax of Scopus and Web of Science while preserving the same conceptual domains. The publication period was restricted to 2000–2025.

No restriction based on age at outcome assessment was applied because eligible outcomes could occur in adulthood following dietary exposure during childhood. Likewise, longitudinal design terms such as “cohort,” “longitudinal,” or “follow-up” were not mandatory components of the main search strategy in order to maximize sensitivity; temporality was instead assessed during study selection.

Reference lists of included studies and relevant systematic reviews were also screened to identify additional potentially eligible sources.

The complete database-specific search strategies, including all search terms, field tags, Boolean operators, database-specific adaptations, applied limits (English language; publication years 2000–2025; human studies), the date of the last search, and the number of records retrieved from each database, are provided in Table S1, reported according to the PRISMA-S extension. All records screened, with their source database, identifiers, screening decision, selection stage, and reason for exclusion, are listed in Table S6, and the classification, characteristics, non-retrieved reports, and full-text exclusions are provided in Tables S2–S5.

### 2.6. Study Selection

All retrieved records were exported and merged into a single review database, and duplicate records were removed before screening.

Study selection was conducted in two stages: title and abstract screening followed by full-text eligibility assessment. At both stages, records were assessed against the predefined eligibility criteria.

At the full-text stage, reasons for exclusion were recorded using standardized categories, including absence of an eligible childhood dietary exposure; exposure not preceding the outcome; absence of an eligible immune and inflammatory or life-course outcome; therapeutic diet initiated only after diagnosis; cross-sectional design without temporal follow-up; ineligible population, publication type, or publication period; and other prespecified reasons.

All records were managed in a single review database, in which duplicates were identified sequentially by digital object identifier (DOI), exact normalized title, and high-similarity title matching followed by manual audit. Title and abstract screening and full-text assessment were conducted between 22 August and 12 September 2026 using a single set of predefined operational eligibility rules. Records whose eligibility could not be determined unequivocally from the title and abstract were retained for full-text assessment, and every excluded record was assigned one primary reason from a standardized list.

Screening and full-text assessment were assisted by a large language model, Chat GPT-6 model (OpenAI, San Francisco, CA, USA), which was provided with the eligibility rules and with the bibliographic record or full text of each report. Each AI-assisted decision was reviewed and confirmed by one author (R.-N.V.), and uncertain decisions were resolved by discussion among the authors. Before the final analysis, 51 exclusion decisions were re-reviewed, of which 12 were reversed. Independent dual screening of all records by two human reviewers and formal systematic validation of AI-assisted decisions against an independently established reference set were not performed. All decisions, retrieval statuses, and exclusion reasons were reconciled in a single master database before the final counts were derived. For a subset of records, the title/abstract screening status was reconstructed retrospectively during this reconciliation, rather than retrieved as a contemporaneously documented decision; these records are labelled in Table S6, and each reconstructed decision was checked against the predefined eligibility criteria using the available title and abstract and confirmed by the responsible author.

Full texts were sought through PubMed Central, publisher websites, and the DOI and PubMed records; reports still lacking a full text after the first round were searched for again in a second reconciliation step. Reports not available through these sources were additionally sought through institutional library access and requests to corresponding authors, as applicable. Only a complete article was accepted as evidence of retrieval. Reports for which no verified full-text source could be identified were recorded as not retrieved (Table S4) and were not counted as assessed or included. Each report excluded at full-text assessment was recorded with a single primary reason (Table S5).

The study-selection process was summarized using a PRISMA-ScR flow diagram included in the main manuscript (Figure 1).

### 2.7. Data Charting

A standardized data-charting form was developed specifically for this review and pilot-tested using studies representative of the principal outcome domains.

For each included study, the following information was charted: author; year of publication; country; cohort or study name; study design; sample size; participant characteristics; health status at baseline; type of dietary exposure; dietary assessment method; age at exposure; developmental exposure window; exposure duration or frequency; outcome category; specific outcome; age at outcome assessment; follow-up duration; immune and inflammatory biomarkers; microbiome or microbial metabolite measures; intestinal barrier markers, where available; proposed mechanistic pathway; effect estimates; direction of association; statistical significance; dose–response findings; sex-specific findings; principal confounders; and confirmation of temporality. The main charted characteristics of each included study are reported in Table S3.

The relationships among age at dietary exposure, age at outcome assessment, and duration of follow-up were explicitly charted for each included source.

Childhood exposure windows were categorized as 0–2 years, 2–5 years, 6–12 years, 13–18 years, or repeated/multiple developmental windows.

### 2.8. Outcome Classification

Included evidence was organized into eight predefined outcome domains:

1. Allergic and atopic outcomes;
2. Autoimmune and immune-mediated outcomes;
3. Gastrointestinal and gut-mediated outcomes;
4. Inflammatory and immunological biomarkers;
5. Immunometabolic outcomes;
6. Hepatic and immunometabolic liver outcomes;
7. Neurocognitive and neuroinflammatory outcomes;
8. Endocrine–immunometabolic outcomes.

For classification purposes, allergic and atopic outcomes included asthma, wheezing, eczema, allergic rhinitis, food allergy, and allergic sensitization. Autoimmune and immune-mediated outcomes included diagnosed autoimmune or immune-mediated disorders. Gastrointestinal and gut-mediated outcomes encompassed gastrointestinal diseases, intestinal barrier-related measures, and gut microbial outcomes. Inflammatory and immunological biomarkers included immunoglobulins, cytokines, inflammatory markers, and immune-cell measurements. Immunometabolic outcomes included metabolic phenotypes investigated in relation to immune or inflammatory processes. Hepatic and immunometabolic liver outcomes comprised liver-related clinical, biochemical, and inflammatory or metabolic phenotypes. Neurocognitive and neuroinflammatory outcomes included cognitive, neurodevelopmental, and neurological inflammatory endpoints. Endocrine–immunometabolic outcomes encompassed endocrine-related phenotypes investigated in relation to metabolic or immune-inflammatory processes.

Studies were assigned to domains according to the outcomes actually assessed. Where relevant, overlapping conditions, such as celiac disease and inflammatory bowel disease, were considered across both autoimmune/immune-mediated and gastrointestinal domains, as appropriate. The eight outcome domains were predefined to ensure that the search and selection process captured all relevant outcomes. The amount of evidence identified differed markedly between domains. Results are therefore presented in proportion to the evidence available, and the limited representation of a domain is interpreted as an evidence gap rather than as evidence of absence of association.

### 2.9. Classification of Mechanistic Evidence

Each included study was assigned to one of three mutually exclusive evidence tiers according to the type of biological information it reported, and not according to its methodological quality. (1) Direct immunometabolic evidence: within the same study, a metabolic or nutritional pathway (e.g., fatty-acid profiles, human-milk bioactive components, micronutrient status, gut-microbiome composition, or microbial or systemic metabolites) was measured together with an immune or inflammatory component (e.g., total or allergen-specific immunoglobulin (Ig) E, IgA, cytokines, immune-cell phenotypes, or markers of immune tolerance). (2) Mechanistically indirect evidence: the study measured an intermediate biological pathway linking diet with the outcome on only one side of the immunometabolic interface, that is, an immune or sensitization measure (e.g., IgE or skin-prick sensitization) without a concurrently measured metabolic or nutritional pathway, or a metabolic, microbial, or human-milk compositional measure without a concurrently measured immune component. (3) Predominantly clinical diet–outcome association: a longitudinal association between dietary exposure and a clinical outcome without measurement of a defined intermediate biological mediator.

Classification followed a stepwise rule applied after full-text review: first, whether a metabolic/nutritional pathway measure and an immune/inflammatory measure were both reported within the same study (direct); if not, whether any intermediate biological measure was reported (indirect); otherwise, the study was classified as predominantly clinical. During data charting, the mechanistic content of each study was recorded using a fixed set of thematic tags, which were mapped to six mechanistic axes: (1) allergic sensitization, IgE/IgA responses, and immune tolerance; (2) lipid and fatty-acid pathways; (3) vitamins, micronutrients, and antioxidants; (4) cytokine signaling and immune-cell programming; (5) gut microbiome and microbial or systemic metabolites; and (6) human-milk oligosaccharides and other milk-derived bioactives. A study could be mapped to more than one axis but to only one tier. Tiers and axes were assigned by one author (R.-N.V.) with the assistance of a large language model and confirmed against the predefined rule; uncertain assignments were resolved by discussion among the authors. The tier, axes, and recorded mechanistic tags of each study are listed in Table S2.

### 2.10. Synthesis of Evidence

Given the anticipated heterogeneity in dietary exposures, developmental windows, study populations, outcome definitions, biomarkers, and follow-up durations, the evidence was synthesized using descriptive evidence mapping and narrative synthesis rather than quantitative meta-analysis. Studies were first summarized according to study design, population characteristics, developmental window of dietary exposure, type of exposure, age at outcome assessment, follow-up duration, and direction of association.

Evidence was subsequently synthesized within the predefined outcome domains and, where sufficient information was available, organized according to the following proposed conceptual pathway, which was used as a framework for mapping the evidence and not as a demonstrated or a priori causal model: Childhood dietary exposure → gut microbiota and microbial metabolites → intestinal barrier → innate/adaptive immune programming → systemic or tissue-specific inflammation → long-term clinical phenotype.

Potential gut–liver, gut–brain, and immune–metabolic–endocrine pathways were considered as complementary life-course mechanisms proposed on the basis of background literature.

To distinguish mechanisms examined directly within individual studies from pathways inferred across studies, each included study was assigned to one of the three evidence tiers defined in Section 2.9. Throughout the review, “immunometabolic programming” is used as a hypothesis-generating concept describing a proposed long-term influence of early diet on immune and metabolic regulation; it does not denote a process demonstrated by the included studies.

Steps of the conceptual pathway that were supported only by convergence across different studies, or by literature external to the included studies, are described in this review as proposed rather than demonstrated.

Particular attention was given to the consistency and direction of reported associations, critical developmental windows, persistence of outcomes into adulthood, dose–response relationships, sex-specific effects, and major evidence gaps.

The three evidence tiers were mutually exclusive, so that each of the 403 included studies was assigned to a single tier. In contrast, exposure categories, outcome domains, and mechanistic axes were not mutually exclusive: a study could be mapped to more than one axis and was counted once within each axis to which it was mapped. No weighting or adjustment for overlap was applied. Study counts across axes or categories were therefore not summed, and percentages were calculated using the 403 unique studies (or the 179-study principal mechanistic evidence base, where stated) as denominators.

### 2.11. Critical Appraisal

A formal risk-of-bias assessment was not undertaken because the primary objective of this scoping review was to map the extent, characteristics, and gaps of the available evidence rather than to estimate a pooled effect.

Study design and major methodological characteristics were nevertheless charted and considered when interpreting the evidence.

## 3. Results

### 3.1. Study Selection

Database searching yielded 2039 unique records after removal of duplicates (MEDLINE via PubMed, *n* = 504; Scopus, *n* = 1162; Web of Science Core Collection, *n* = 373); no additional records were identified through reference-list screening. Of these, 1504 (73.8%) were excluded at title and abstract screening, and 535 reports were sought for full-text retrieval. A total of 120 reports (22.4%) could not be retrieved because no verified full-text source could be identified (Table S4); 73 of these had no PubMed identifier, and 111 were indexed only in Scopus or Web of Science. The remaining 415 reports underwent full-text assessment, of which 12 (2.9%) were excluded: no eligible outcome (*n* = 6), therapeutic diet initiated after diagnosis (*n* = 2), no prospective exposure–outcome sequence (*n* = 2), and maternal or prenatal exposure only (*n* = 2) (Table S5). The remaining 403 studies were included in the review. The study-selection process is summarized in the PRISMA-ScR flow diagram (Figure 1).

### 3.2. General Characteristics and Structure of the Evidence Base

The 403 included studies comprised a heterogeneous body of longitudinal pediatric nutrition research in which dietary exposures occurred between birth and 18 years of age and outcomes were assessed at subsequent developmental time points. Prospective and birth-cohort studies predominated, complemented by randomized dietary interventions and other longitudinal observational designs.

Dietary exposures included breastfeeding and human-milk composition, formula feeding, complementary feeding and timing of allergenic-food introduction, individual foods and nutrients, fatty-acid exposure, vitamin and micronutrient intake or supplementation, prebiotic and probiotic interventions, dietary diversity, and broader measures of dietary patterns and diet quality.

Allergic and atopic phenotypes were the most extensively represented clinical outcomes and included asthma and wheeze, eczema or atopic dermatitis, food allergy, allergic rhinitis, and allergic sensitization. Mechanistic measurements included IgE and IgA responses, cytokines, immune-cell phenotypes, human-milk bioactive components, fatty-acid profiles, gut-microbiome composition, microbial and systemic metabolites, and markers of immune tolerance.

The distribution of the 403 included studies by study design, region, developmental exposure window, follow-up duration, and primary outcome category is shown in Table 1. Prospective or birth-cohort studies predominated (236 studies; 58.6%), followed by randomized dietary interventions, including long-term follow-up trials (87; 21.6%), and case–control studies (32; 7.9%). Most studies were conducted in Europe (211; 52.4%), followed by Asia (75; 18.6%), North America (51; 12.7%), and Oceania (31; 7.7%), whereas South America (10; 2.5%) and Africa (4; 1.0%) were poorly represented. Dietary exposure occurred within the first two years of life in 368 studies (91.3%), whereas exposures at 2–5 years (*n* = 11), 6–12 years (*n* = 13), and 13–18 years (*n* = 1) were rarely examined. Allergic and atopic outcomes were the primary outcome category in 398 studies (98.8%). Direct immunometabolic evidence derived almost exclusively from cohort studies and randomized trials conducted in infancy.

Each study was counted once within each characteristic, in its primary category. Exposure window refers to the age at which the dietary exposure was assessed; studies with exposure assessed in more than one window are classified as “Repeated/multiple windows”. Follow-up duration was defined as the interval between the dietary exposure and the latest outcome assessment. Percentages use the 403 included studies as the denominator. Evidence tiers are defined in Section 2.9; study-level data are provided in Tables S2 and S3.

For the mechanistic synthesis, studies were classified according to the degree of immunometabolic evidence provided. Thirty studies simultaneously assessed a metabolic or nutritional pathway together with an immune or inflammatory component and were categorized as direct immunometabolic evidence. A further 149 studies provided mechanistically indirect evidence linking diet with an intermediate biological pathway, such as the microbiome, nutrient metabolism, human-milk composition, or related biological programming. The remaining 224 studies predominantly reported longitudinal clinical diet–outcome associations without measurement of a sufficiently defined immunometabolic mediator. The classification assigned to each included study is provided in Table S2.

Accordingly, 179 studies constituted the principal mechanistic evidence base, while the wider clinical literature provided information regarding the direction and persistence of longitudinal diet–outcome associations. The three evidence tiers are mutually exclusive (30 + 149 + 224 = 403 unique studies). By contrast, exposure categories, outcome domains, and mechanistic axes were not mutually exclusive, and a single study could be mapped to more than one of them; counts within these categories should therefore not be summed.

General characteristics of the included evidence are summarized in Table 2. The main characteristics of each included study—author and year, country, study design, sample size, participant characteristics, dietary exposure, age at exposure, follow-up duration, outcomes assessed, and principal findings—are provided in Table S3, which uses the same study identifiers as the evidence classification in Table S2.

### 3.3. Developmental Timing of Dietary Exposure

The evidence was strongly concentrated in infancy and early childhood, particularly in relation to breastfeeding, formula composition, complementary feeding, and allergenic-food introduction. Studies conducted during later childhood more frequently assessed individual nutrients, food groups, dietary diversity, and broader dietary patterns.

Associations involving fatty acids, vitamins, complementary feeding, and allergenic-food introduction varied according to age at exposure, underlying susceptibility, and age at outcome assessment. The direction of association therefore differed across dietary exposures and developmental windows.

Follow-up duration was <5 years in 173 studies (42.9%), 5–10 years in 101 (25.1%), and >10 years in 30 (7.4%), and outcomes were assessed in adulthood in 14 studies (3.5%) (Table 3). Evidence on long-term associations beyond childhood therefore derives from 44 studies (10.9%), including 5 of the 30 studies providing direct immunometabolic evidence; these studies document the timing of associations and do not, by themselves, demonstrate persistent biological changes. Most of the evidence thus reflects short-term or intermediate associations, which, according to the predefined rule, do not by themselves indicate persistent biological programming.

Follow-up duration was defined as the interval between the dietary exposure and the latest outcome assessment reported. Outcomes assessed <5 years after exposure were interpreted as short-term associations, 5–10 years as intermediate, and >10 years or in adulthood as long-term associations relevant to, but not demonstrative of, persistence. Evidence tiers are defined in Section 2.9.

For synthesis, developmental timing was classified according to the predefined exposure windows of 0–2 years, 2–5 years, 6–12 years, 13–18 years, and repeated or multiple developmental windows.

### 3.4. Allergic Sensitization, IgE/IgA Responses, and Immune Tolerance

Allergic sensitization and immune-tolerance outcomes represented the largest mechanistically relevant component of the evidence base. A total of 156 studies were mapped to IgE, IgA, allergic sensitization, or tolerance-related pathways. Of these, 25 provided direct immunometabolic evidence, 120 were classified as mechanistically indirect, and 11 were predominantly clinical studies in which sensitization-related outcomes were central to the analysis.

The most frequently examined exposures were breastfeeding or human-milk exposure, formula or cow’s-milk exposure, complementary feeding and allergenic-food introduction, followed by dietary patterns or specific foods, fatty acids and micronutrients, and probiotic or prebiotic interventions.

Sensitization outcomes were assessed more frequently than isolated immunoglobulin concentrations, although total and allergen-specific IgE and IgA-related outcomes were also represented.

Early oral exposure to allergenic foods was examined in several randomized and prospective studies. In studies of egg introduction, repeated early exposure was associated with tolerance-related immunological changes, including increased allergen-specific IgG4, while a substantial proportion of high-risk infants were already sensitized before or at the time of first exposure [12].

### 3.5. Lipid Immunometabolism and Fatty-Acid Pathways

Twenty studies were mapped to lipid immunometabolism, although two provided peripheral rather than central lipid-metabolic evidence. The core literature examined human-milk fatty-acid composition, long-chain polyunsaturated fatty acids (PUFA), *n*-3 and *n*-6 PUFA exposure, fish consumption, and genetic variation affecting fatty-acid metabolism.

The direction of association was heterogeneous. Higher intake of long-chain n-3 fatty acids or fish was associated with lower allergic or respiratory risk in some cohorts, whereas other prospective studies and randomized interventions reported null findings for asthma, eczema, or atopy.

Several studies identified effect modification by metabolic or genetic factors. In one longitudinal analysis, higher eicosapentaenoic acid (EPA) and docosahexaenoic acid (DHA) intake was associated with lower incident asthma risk among carriers of a specific fatty acid desaturase (FADS) variant, whereas no comparable association was observed in children with the alternative genotype [13]. Interactions between n-3 PUFA exposure and genetic variation in the Dipeptidyl Peptidase Like 10 (DPP10) region were also reported in relation to childhood atopy [14].

Studies of human-milk fatty-acid composition produced similarly heterogeneous clinical findings. Breastfeeding was associated with selected favorable respiratory outcomes in some cohorts, but variation in the fatty-acid composition of human milk did not consistently account for these associations.

Direct mechanistic studies also linked lipid exposures to inflammatory pathways. Long-chain saturated fatty acids in human milk were associated with inflammatory immune-cell responses, including group 3 innate lymphoid cells (ILC3)-related signaling [15], while other studies related fatty-acid metabolism to sensitization and atopic phenotypes.

Overall, lipid-related associations varied according to pathway, host characteristics, and developmental context.

### 3.6. Vitamins, Micronutrients, and Antioxidant Metabolism

Fifteen studies were mapped to vitamin, micronutrient, or antioxidant-related pathways. Principal exposures included vitamins A and D, antioxidant nutrients, fruit- and vegetable-related antioxidant intake, and composite dietary antioxidant capacity.

Higher beta-carotene intake and greater total dietary antioxidant capacity were associated in several prospective analyses with lower allergic sensitization, lower IgE concentrations, or lower allergic asthma risk [16,17].

By contrast, findings for isolated vitamin supplementation were heterogeneous. Vitamin A supplementation was associated with null, favorable, or adverse outcomes depending on dose, sex, timing, and study population [18,19]. Vitamin D exposure showed similarly variable findings, with some studies suggesting differences according to developmental timing and combined prenatal and postnatal exposure [20].

Associations therefore differed between isolated supplementation and broader dietary antioxidant exposures.

### 3.7. Cytokine Signaling and Immune-Cell Programming

Thirteen studies addressed cytokine signaling, immune-cell programming, or tolerance-related pathways (2 classified as direct and 11 as mechanistically indirect evidence; Table 4). Human milk represented the principal dietary context in this group.

Measured bioactive immune components included transforming growth factor beta (TGF-β), interleukin (IL)-10, IL-1β, soluble CD14, IgA, and other cytokines or chemokines. Several prospective analyses related variation in these milk-derived factors to subsequent eczema, atopy, allergic manifestations, or maturation of the infant immune system [21].

One longitudinal comparison of breastfeeding and formula feeding reported a more anti-inflammatory cytokine profile in breastfed infants, characterized by lower circulating tumor necrosis factor alpha (TNF-α) and IL-2 and higher TGF-β during infancy [22].

Interactions between dietary and environmental exposures were also reported. In one childhood supplementation study, fish-oil exposure modified associations between traffic-related air pollution, allergen sensitization, and allergen-specific IL-5 responses [23].

### 3.8. Gut Microbiome, Metabolomics, and Microbial Metabolites

Fourteen studies were mapped to microbiome or metabolomic pathways.

Breastfeeding was associated with differences in early microbial colonization, including enrichment of bifidobacterial taxa. In one longitudinal cohort, enrichment of Bifidobacterium longum subsp. infantis was associated with attenuation of antibiotic-related microbiome disruption and subsequent childhood asthma risk [24].

Formula feeding was associated with distinct metabolic signatures. Longitudinal metabolomic analysis identified urinary metabolites associated with both formula exposure and subsequent milk sensitization, including microbial-derived metabolites and compounds related to amino-acid metabolism [25].

Dietary diversity was also examined in relation to microbial development. In a longitudinal cohort, lower dietary diversity during infancy was associated with lower gut microbial richness, which was subsequently associated with food-allergy development [26,27].

Prebiotic and probiotic interventions produced changes in microbial composition or functional capacity in several studies, although changes in overall community structure were not consistently observed.

Direct measurements of individual short-chain fatty acids were not sufficiently consistent across the included studies to support a separate SCFA-specific evidence category.

### 3.9. Human-Milk Oligosaccharides and Other Milk-Derived Bioactives

Ten studies focused on human-milk oligosaccharides (HMOs), other milk-derived bioactive compounds, or prebiotic oligosaccharides intended to reproduce selected functional properties of human milk.

Human-milk oligosaccharide composition was prospectively associated with eczema and food-allergy outcomes, with associations differing according to the individual oligosaccharide and feeding context. Concentrations of 2′-fucosyllactose and 6′-sialyllactose, for example, showed different relationships with subsequent allergic outcomes [28].

Other human-milk constituents, including secretory IgA, lactoferrin, TGF-β, and milk-associated microbial communities, were examined in relation to neonatal immune maturation and subsequent allergic phenotypes [29].

Trials of formula supplemented with prebiotic oligosaccharides provided complementary evidence. In some studies, galacto- and fructo-oligosaccharide supplementation increased fecal bifidobacteria and lactobacilli toward profiles resembling those observed during breastfeeding and was accompanied by fewer allergic manifestations [30]. Clinical effects, however, were not uniform across interventions.

### 3.10. Diet Quality, Dietary Diversity, and Broader Dietary Patterns

Seventeen studies specifically assessed diet quality, dietary diversity, healthy or Western-type dietary patterns, Mediterranean-type dietary exposure, or related composite dietary constructs.

Dietary diversity showed the most consistent direction of association within this group. Several prospective cohorts reported inverse associations between greater diversity of complementary foods during infancy and subsequent food allergy or broader allergic disease. In one cohort, infants with the highest weighted dietary-diversity score at 9 months had substantially lower odds of food allergy at 18 months than those with the lowest diversity [31].

Lower dietary diversity was also associated with reduced microbial richness and subsequent food-allergy development [27].

Findings for broader dietary-pattern and diet-quality scores were more heterogeneous. Mediterranean-type and healthier dietary patterns were associated with favorable outcomes in some studies, whereas other cohorts reported no clear relationship between maternal or infant dietary-pattern scores and food allergy [32,33]. Western-type dietary patterns were associated with more frequent respiratory symptoms in selected prospective cohorts, although some associations were partly explained by overall energy intake [34].

Evidence regarding ultra-processed food exposure was limited and did not consistently demonstrate an independent association with allergic or respiratory outcomes within the longitudinal literature included in this review [35].

The whole-diet evidence is summarized in Table 4.

### 3.11. Proposed Integrated Immunometabolic Pathways

Across the included evidence, six recurrent and overlapping mechanistic axes were identified:

1. Allergic sensitization, IgE/IgA responses, and immune tolerance;
2. Lipid and fatty-acid immunometabolism;
3. Vitamin, micronutrient, and antioxidant metabolism;
4. Cytokine signaling and immune-cell programming;
5. Gut microbiome and microbial or systemic metabolites;
6. Human-milk oligosaccharides and other milk-derived bioactive signals.

Diet quality and dietary diversity intersected with several of these axes as broader dietary exposures.

Across studies, dietary exposure was associated with combinations of metabolic substrates and bioactive signals, microbial ecology and metabolite production, cytokine and immune-cell responses, and subsequent sensitization, inflammatory, or clinical outcomes.

Relatively few individual studies measured the complete sequence within the same population. Most assessed selected components, such as diet and clinical allergy, diet and the microbiome, or diet and immune biomarkers. The mechanistic evidence therefore arose predominantly from convergence across complementary studies rather than complete pathway assessment within individual longitudinal cohorts.

The six principal mechanistic axes are summarized in Table 5. Mapping to the six axes was not mutually exclusive. The 228 axis assignments shown in Table 5 (60 direct, 154 indirect, and 14 predominantly clinical) derive from the 403 unique included studies and therefore exceed the number of unique studies. For example, the 60 direct-evidence assignments arose from 30 unique studies, corresponding to an average of two axes per direct-evidence study. The axes assigned to each study are listed in Table S2.

Table 6 distinguishes, for each step of the proposed pathway, the findings directly observed within individual included studies from the steps that are proposed on the basis of convergence across studies or of literature external to the included studies. Only 30 of the 403 included studies (7.4%) provided relatively direct immunometabolic evidence, and even these studies generally assessed selected components of the pathway rather than the complete sequence.

### 3.12. Evidence Gaps

Allergic and atopic phenotypes accounted for most of the mapped evidence. A total of 156 of the 403 included studies (38.7%) were mapped to the allergic sensitization, IgE/IgA, and immune-tolerance axis, and 145 of these (25 direct and 120 indirect) contributed mechanistic information, corresponding to 81.0% of the 179 studies in the principal mechanistic evidence base. Each of the remaining five mechanistic axes comprised between 10 and 20 studies (Table 5). Studies addressing autoimmune and immune-mediated, gastrointestinal, hepatic, neurocognitive, and endocrine–immunometabolic outcomes were less extensively represented and did not form distinct mechanistic axes in the present synthesis. Mechanistic studies of dietary exposures in middle childhood and adolescence were also comparatively scarce. These patterns indicate gaps in the available longitudinal evidence and should not be interpreted as evidence of absence of association.

The evidence was concentrated in infancy and early childhood, particularly around breastfeeding, formula exposure, complementary feeding, allergenic-food introduction, and allergic disease. Mechanistic studies of dietary exposures during later childhood and adolescence were comparatively less represented. Specifically, only 13 studies examined dietary exposures at 6–12 years and a single study at 13–18 years; only 14 studies originated from South America or Africa, and 398 of the 403 studies (98.8%) addressed allergic and atopic outcomes as the primary outcome category (Table 1).

Few studies simultaneously assessed dietary exposure, microbiome composition, microbial or systemic metabolites, immune-cell phenotypes, cytokines, and subsequent clinical outcomes. Consequently, the complete sequence linking dietary exposure with metabolic or microbial changes, immune programming, and persistent clinical outcomes was rarely evaluated within the same longitudinal population.

Genetic and sex-specific effect modification was reported in several mechanistic domains, particularly lipid metabolism, but was not routinely evaluated.

Finally, substantial heterogeneity was present in dietary assessment, biomarker selection, outcome definitions, follow-up duration, and developmental timing, limiting direct comparisons across studies.

## 4. Discussion

### 4.1. Principal Findings

This scoping review mapped a large and heterogeneous body of longitudinal evidence examining how dietary exposures during childhood relate to subsequent immune and inflammatory health. Although the review encompassed a broad range of immunoinflammatory and life-course outcomes, the evidence was concentrated primarily in allergic and atopic phenotypes and in exposures occurring during infancy and early childhood.

Of the 403 included studies, only 30 (7.4%) provided relatively direct immunometabolic evidence and 149 (37.0%) provided mechanistically indirect evidence, together forming a principal mechanistic evidence base of 179 studies; the remaining 224 studies (55.6%) predominantly reported longitudinal clinical diet–outcome associations.

The principal pathways involved allergic sensitization and immune tolerance, lipid and fatty-acid metabolism, micronutrient and antioxidant pathways, cytokine signaling, gut microbial ecology and microbial metabolites, and human-milk-derived bioactive signals. Dietary diversity and overall diet quality intersected with several of these pathways rather than representing isolated biological mechanisms.

The scope of these findings should be read accordingly. Allergic and atopic outcomes accounted for the large majority of the mechanistic evidence (145 of the 179 studies in the principal mechanistic evidence base were mapped to the sensitization, IgE/IgA, and tolerance axis), whereas evidence on autoimmune, gastrointestinal, hepatic, neurocognitive, and endocrine–immunometabolic outcomes, and on dietary exposures in middle childhood and adolescence, was more limited. The framework discussed here is therefore best supported for early-life dietary exposures and allergic and atopic phenotypes, and its applicability to other immunological outcomes and to later developmental windows remains to be established.

A consistent feature across exposure categories was the absence of a simple relationship in which individual foods or nutrients could be classified as uniformly protective or harmful. Associations varied according to developmental timing, host susceptibility, genetic background, baseline immune state, feeding context, and the biological pathway under investigation. This heterogeneity was particularly evident for fatty acids, vitamin supplementation, breastfeeding, and allergenic-food introduction.

The findings are therefore compatible with a proposed model of context-dependent immunometabolic programming, in which dietary exposure may interact with microbial, metabolic, and immune processes during development; only 30 of the 403 studies (7.4%) provided direct immunometabolic evidence, and none measured the complete sequence from diet to clinical outcome. Most studies, however, assessed only selected components of this proposed pathway. The mechanistic interpretation of the review should consequently be understood as convergence across complementary studies rather than as direct demonstration of a complete causal sequence within individual longitudinal cohorts.

### 4.2. Developmental Timing, DOHaD, and Early-Life Immunometabolic Programming

The concentration of evidence in infancy and early childhood is biologically relevant. During this period, intestinal microbial colonization, epithelial maturation, establishment of immune tolerance, and adaptive immune development occur simultaneously and remain highly responsive to environmental exposures. Breastfeeding, formula composition, complementary feeding, dietary diversification, and first exposure to food antigens therefore occur within a particularly plastic developmental environment. Moreover, only 44 of the 403 included studies (10.9%) assessed outcomes more than 10 years after exposure or in adulthood, so that the persistence of diet-related immune differences beyond childhood remains largely untested.

These observations are consistent with the Developmental Origins of Health and Disease (DOHaD) framework [39], which proposes that exposures during critical developmental windows may modify biological trajectories and later susceptibility to disease. Within the present review, nutritional immunometabolism represents one plausible mechanism through which such programming may occur.

Importantly, earlier exposure was not uniformly beneficial. Associations varied according to the dietary factor, developmental window, genetic background, and immunological state of the child. This was especially apparent in studies of allergenic-food introduction, vitamins, and fatty acids.

Developmental timing may therefore represent a biological determinant of nutritional associations in addition to being a descriptive feature of study design. At the same time, the current evidence remains disproportionately concentrated in early life, leaving middle childhood and adolescence substantially less characterized from a mechanistic perspective. Conclusions regarding these later developmental windows cannot be drawn from the present evidence and should not be extrapolated from findings obtained in infancy.

### 4.3. Sensitization and Tolerance as Central Immunometabolic Outcomes

Allergic sensitization was the most extensively represented mechanistic outcome in the review. The large number of studies assessing IgE, IgA, skin-prick sensitization, food allergy, and related immune responses is compatible with the view that the development of tolerance may represent a major biological interface between childhood nutrition and subsequent immune disease.

The findings are compatible with the view that sensitization is not determined solely by antigen exposure. Rather, the response to dietary antigens appears to depend on the metabolic, microbial, and immunological environment in which exposure occurs. Human-milk composition, fatty-acid metabolism, microbial colonization, micronutrient status, and complementary feeding practices may all contribute to this context.

Studies of early egg exposure illustrate this complexity. Repeated oral exposure was associated with tolerance-related responses, including increased allergen-specific IgG4, whereas a substantial proportion of high-risk infants had already developed evidence of sensitization at or before first exposure [12]. Associations between allergenic-food introduction and tolerance-related outcomes may therefore differ according to the immunological state present when exposure begins.

These observations are compatible with the possibility that similar dietary exposures are associated with different biological responses depending on the host’s capacity to metabolize or respond to those exposures.

### 4.4. Lipid Immunometabolism: Pathway-Specific and Host-Dependent Effects

The lipid literature provides a clear example of why nutritional immunology cannot be reduced to simple categories of beneficial and harmful nutrients.

Omega-3 fatty acids and fish intake were associated with favorable allergic or respiratory outcomes in selected cohorts, while null findings were common in both observational studies and intervention trials. Part of this variability may reflect differences in host metabolism and genetic susceptibility.

Interactions involving FADS-related fatty-acid metabolism are particularly informative. In some cohorts, higher EPA and DHA intake was associated with lower asthma risk only among children carrying particular genetic variants [13]. Similar interactions were observed between n-3 PUFA exposure and DPP10-region variants in relation to childhood atopy [14].

These observations suggest that identical dietary exposures may produce different biological effects depending on the host’s capacity to metabolize or respond to those exposures. Fatty acids can influence membrane composition, eicosanoid synthesis, transcriptional signaling, immune-cell function, and microbial ecology, and the relative importance of these pathways may vary between individuals.

Human-milk fatty-acid studies reinforce this interpretation. Although breastfeeding was associated with favorable outcomes in some cohorts, variation in human milk PUFA composition did not consistently account for these associations. The immunological associations of breastfeeding are therefore unlikely to be explained by the lipid fraction of human milk alone.

Specific long-chain saturated fatty acids have also been associated with inflammatory ILC3-related responses [15], which is compatible with the possibility that lipid signaling operates directly at the interface between metabolism and immunity.

### 4.5. Micronutrients, Antioxidant Balance, and the Limits of Isolated Supplementation

Micronutrient-related evidence was heterogeneous. Vitamin A and vitamin D exposures were associated with favorable, null, or occasionally adverse outcomes depending on dose, developmental timing, sex, and study population [18,19,20].

These findings argue against the assumption that greater exposure to an immunologically active micronutrient is necessarily beneficial. Nutrients involved in immune regulation operate within tightly controlled biological systems, and both insufficient and excessive exposure may alter normal developmental processes.

Dietary antioxidant exposure showed a somewhat more coherent favorable pattern. Higher beta-carotene intake and greater total dietary antioxidant capacity were associated in several cohorts with lower allergic sensitization, lower IgE concentrations, or reduced allergic asthma risk [16,17].

The contrast between broader dietary antioxidant exposure and isolated supplementation may be biologically meaningful. Foods provide combinations of vitamins, carotenoids, polyphenols, fiber, minerals, and other bioactive compounds that may influence oxidative balance and inflammatory signaling through interacting pathways.

The available evidence therefore supports a distinction between isolated nutrient supplementation and the broader biological context in which nutrients are consumed as part of a dietary pattern.

### 4.6. Cytokine Signaling, Human Milk, and Immune Programming

The cytokine and immune-cell literature provides some of the more direct evidence within the included studies for diet-related differences in immune signaling; whether these differences mediate later clinical outcomes was not established by the studies summarized.

Human milk contains multiple biologically active components capable of interacting with the developing immune system, including TGF-β, IL-10, IL-1β, secretory IgA, soluble CD14, and lactoferrin. Studies included in this review linked variation in several of these components with neonatal immune development and later allergic outcomes.

Comparisons between breastfed and formula-fed infants showed that feeding mode was associated with differences in the systemic cytokine environment. Higher TGF-β and lower pro-inflammatory cytokine concentrations during breastfeeding are compatible with an immune milieu favoring regulated maturation and tolerance [21,22].

Human milk may therefore be considered not only as a nutritional substrate but also as a complex biological signaling system containing immune, microbial, metabolic, and nutritional components.

Nutritional factors may also modify immune responses to non-dietary exposures. The reported interaction between fish-oil supplementation and traffic-related air pollution in relation to allergic sensitization and IL-5 responses suggests that dietary exposure may alter the biological consequences of environmental inflammatory stress, although this was reported in a single supplementation study [23].

### 4.7. The Diet–Microbiome–Metabolite–Immune Axis

The gut microbiome represents an important biological interface between diet and immune development.

Diet determines the substrates available to intestinal microbial communities, while microbial metabolism generates bioactive compounds capable of interacting with epithelial, metabolic, and immune pathways. The included studies showed associations between breastfeeding, formula feeding, dietary diversity, probiotics or prebiotics, and differences in microbial composition or function.

Breastfeeding-related enrichment of bifidobacterial species was particularly notable. In one longitudinal cohort, enrichment of Bifidobacterium longum subsp. infantis was associated with attenuation of antibiotic-related microbiome disruption and subsequent asthma risk [24].

Metabolomic studies provided an additional functional layer. Formula feeding was associated with microbial-derived and amino-acid-related metabolites that were also related to IgE-mediated milk sensitization [25]. Such observations extend interpretation beyond microbial taxonomy toward functional metabolic signatures.

Dietary diversity was similarly associated with microbial ecology. Lower diversity of dietary exposure during infancy was associated with reduced gut microbial richness and subsequent food-allergy development [27].

These observations are compatible with a role for microbial function in addition to taxonomic composition, although the included studies do not permit comparison of their relative contributions. However, direct measurements of specific short-chain fatty acids were not sufficiently represented in the included corpus to support a strong SCFA-specific conclusion.

The broader biological role of SCFAs is supported by mechanistic literature demonstrating effects on immune regulation and regulatory T-cell development [36,37,38], but this external mechanistic evidence should remain distinct from direct findings within the studies included in the present review.

### 4.8. Human-Milk Oligosaccharides and Milk-Derived Bioactive Signaling

Human-milk oligosaccharides illustrate a plausible interface among nutrition, the gut microbiota, and immunity.

HMOs are selectively metabolized by specific members of the gut microbiota and may also interact with mucosal and immune pathways. The included studies showed prospective associations between HMO composition and subsequent eczema or food allergy, although associations were not uniformly favorable across individual oligosaccharides [28].

Associations involving 2′-fucosyllactose and 6′-sialyllactose differed according to feeding context, further emphasizing the composition- and context-dependent nature of milk-derived bioactive signals.

Prebiotic oligosaccharide interventions provided complementary experimental evidence. Formula supplementation with galacto- and fructo-oligosaccharides promoted bifidobacterial and lactobacillary profiles closer to those observed during breastfeeding in several studies and was accompanied by fewer allergic manifestations in some populations [30].

Such interventions, however, reproduce only selected biological functions of human milk. Human milk contains a complex mixture of oligosaccharides, immunoglobulins, cytokines, lactoferrin, fatty acids, microbial components, and other bioactive molecules. Its immunological effects are therefore unlikely to be attributable to any single constituent. Its immunological associations are therefore unlikely to be attributable to any single constituent.

### 4.9. Diet Quality and Dietary Diversity as Candidate Upstream Exposures

The whole-diet evidence suggests that overall dietary characteristics may be relevant to several immunometabolic pathways, although most of the evidence is associative.

Dietary diversity showed the most consistent signal. Greater diversity of complementary foods during infancy was associated with lower risk of food allergy or broader allergic outcomes in several cohorts [26,31,32], and mechanistic evidence linked lower dietary diversity with reduced microbial richness and subsequent food allergy [27]. Among the whole-diet constructs (Table 3), dietary diversity therefore provided the strongest signal, whereas composite diet-quality scores, Western-type patterns, and ultra-processed food exposure showed mixed or limited evidence; antioxidant-rich and fruit- and vegetable-based patterns were compatible with redox-related pathways, and Mediterranean-type patterns with multiple nutritional and microbial pathways, but these links were not directly measured.

Dietary diversity may therefore reflect more than feeding behavior alone. A diverse diet provides a broader range of fermentable substrates, nutrients, lipids, phytochemicals, and food antigens, any of which may contribute to microbial and immune development.

Healthy or Mediterranean-type dietary patterns were associated with favorable outcomes in selected studies, whereas Western-type patterns were linked to more frequent respiratory symptoms in some cohorts. These associations were not uniform, and dietary-pattern definitions differed substantially across studies.

The current evidence does not support a single universal dietary score as a validated predictor of allergic disease. Instead, diet quality may operate as an upstream exposure that is plausibly linked to several pathways, including microbial diversity, oxidative balance, lipid signaling, nutrient adequacy, and inflammatory regulation.

### 4.10. A Proposed Integrated Model of Nutritional Immunometabolism

Taken together, the mapped evidence is compatible with a proposed integrated model in which childhood dietary exposure interacts with metabolic, microbial, and immune processes during development.

A simplified conceptual sequence, in which only some steps were directly observed within the included studies (Table 4), can be represented as:

Dietary exposure → metabolic and bioactive signals → microbiome and metabolome → immune signaling and tolerance → sensitization or inflammatory phenotype → clinical outcome.

This sequence is a hypothesis-generating framework and should not be interpreted as a demonstrated causal chain. It should also not be interpreted as strictly linear. Bidirectional and overlapping interactions are likely. Lipids may influence both immune-cell signaling and microbial ecology; microbial metabolites may modify immune differentiation; immune activation may alter metabolic demand; and host genetics may modify nutrient metabolism.

A network model therefore provides a more appropriate representation of nutritional immunometabolism than a single-pathway model.

Four candidate modifiers emerged across the literature: developmental timing, host genetics, baseline immune state, and the broader dietary context. These modifiers may offer a plausible, but untested, explanation for why apparently similar dietary exposures are associated with favorable, neutral, or adverse outcomes in different populations.

### 4.11. Evidence Gaps and Priorities for Future Research

The principal limitation of the existing evidence is the fragmentation of mechanistic measurement.

Only a minority of studies simultaneously assessed diet, microbial composition, metabolites, immune-cell phenotypes, inflammatory mediators, and subsequent clinical outcomes. Most captured only one or two stages of the proposed pathway.

Future longitudinal studies should integrate repeated dietary measurements with microbiome profiling, metabolomics, immune phenotyping, cytokine assessment, intestinal-barrier markers, genetic information, and standardized clinical outcomes.

Repeated measurements are particularly important because a single dietary assessment may not adequately characterize exposure during developmental periods in which feeding practices and dietary patterns change rapidly.

Mechanistic research should also place greater emphasis on functional microbial outputs rather than taxonomy alone. Metabolomics, lipidomics, and targeted analysis of microbial-derived metabolites may help clarify the biological processes connecting diet with immune phenotype.

Sex-specific and genotype-specific analyses should become more common, particularly in light of the effect modification observed in lipid-related pathways.

Finally, middle childhood and adolescence remain less mechanistically characterized than infancy and early childhood. Future studies should determine whether these periods represent additional windows during which nutrition may modify established immune and metabolic trajectories.

Studies from South America, Africa, and other under-represented regions, and studies addressing autoimmune, gastrointestinal, hepatic, neurocognitive, and endocrine–immunometabolic outcomes, are also needed, as these settings and outcome domains are almost absent from the current longitudinal evidence (Table 1).

### 4.12. Strengths and Limitations

A major strength of this review is its life-course approach, in which eligibility was defined according to the timing of dietary exposure rather than age at outcome assessment. This allowed childhood nutrition to be evaluated in relation to outcomes occurring at later developmental stages.

A second strength is the distinction between direct immunometabolic evidence, mechanistically indirect evidence, and predominantly clinical diet–outcome associations. This classification helps avoid treating all longitudinal associations as equivalent evidence of immune programming.

The review also integrates allergic and clinical outcomes with microbial, metabolic, cytokine, nutrient, and milk-bioactive pathways, allowing mechanistic relationships to be considered across literatures that are often examined separately.

Several limitations should nevertheless be acknowledged.

Language and selection bias. Only English-language studies were eligible, and the search was limited to MEDLINE via PubMed, Scopus, and Web of Science Core Collection. Relevant longitudinal studies published in other languages or indexed elsewhere may therefore have been missed, introducing potential language and publication bias. In addition, 120 of the 535 reports sought for retrieval (22.4%) could not be obtained in full text and were therefore not assessed; these reports were more often published in subscription journals and lacked a PubMed identifier, and their exclusion may have introduced selection bias and limited the completeness of the evidence base (Table S4).

Methodological heterogeneity. The breadth of the review resulted in substantial heterogeneity in dietary assessment methods, exposure definitions, study populations, biomarkers, follow-up duration, and outcome ascertainment, which limited direct comparison across studies and precluded quantitative synthesis. Dietary exposure was frequently assessed through parental questionnaires, food-frequency questionnaires, dietary records, or self-report, all of which are susceptible to measurement error. 

Residual confounding. Childhood diet is closely associated with parental health, socioeconomic circumstances, family history, environmental exposures, antibiotic use, delivery mode, adiposity, and other factors that may independently influence immune outcomes. Residual confounding cannot be excluded, particularly in observational studies.

AI-assisted selection. Screening, full-text eligibility assessment, and evidence classification were assisted by a large language model applying predefined rules. Each AI-assisted decision was reviewed by one author, and uncertain cases were discussed among the authors. Before finalization, 51 exclusion decisions were re-reviewed, of which 12 were reversed. However, independent dual screening of all records by two human reviewers and formal systematic validation of AI-assisted decisions were not performed. Accordingly, some degree of selection or classification error cannot be excluded. 

Scope and review-level limitations. The evidence was concentrated in infancy and early childhood and in allergic and atopic outcomes, which limits generalizability to other immunological outcomes and to middle childhood and adolescence. The protocol was not prospectively registered, and a formal risk-of-bias assessment was not undertaken. The findings therefore characterize the extent, structure, and direction of the literature rather than providing a graded estimate of causal certainty. The proposed mechanistic pathways should therefore be interpreted cautiously. Although convergence across studies supports biological plausibility, few individual studies demonstrated the complete sequence from dietary exposure through microbial or metabolic mediation to immune programming and later clinical outcomes.

Associations observed after extended follow-up indicate that childhood dietary exposures may be associated with outcomes at later developmental stages, but they do not establish persistent biological programming. Long-term associations, mechanistic plausibility, and demonstrated biological persistence should be distinguished: the first describes the timing of an observed association, the second reflects a biologically plausible interpretation supported by convergent evidence, and the third requires direct evidence that the relevant biological changes persist over time. In this review, persistence remains insufficiently demonstrated, as only 44 of 403 studies (10.9%) had follow-up exceeding 10 years (*n* = 30) or assessed outcomes in adulthood (*n* = 14) (Table 3); the remaining evidence describes short-term or intermediate associations.

### 4.13. Implications for Nutritional Immunology and Clinical Practice

The findings do not support highly specific dietary prescriptions for the prevention of individual allergic or immune-mediated diseases. The variability observed across studies of fatty acids, vitamins, breastfeeding, complementary feeding, and broader dietary patterns indicates that nutritional effects cannot be generalized across all children or developmental periods.

Early-life diet can be considered a potential developmental exposure interacting with microbial ecology, metabolic signaling, and immune maturation rather than solely a source of energy and nutrients; however, this interpretation rests mainly on early-life exposures and on allergic and atopic outcomes, and it cannot be extended to other immune-mediated outcomes or to later developmental windows on the basis of the present evidence.

Dietary diversity and overall diet quality may be particularly relevant because they may plausibly relate to multiple biological pathways simultaneously. At the same time, responses to a given dietary exposure may differ according to developmental stage, baseline immune state, genetic background, metabolic capacity, and the wider dietary context.

From a clinical perspective, the present evidence is therefore more compatible with an emphasis on nutritionally adequate, diverse, and developmentally appropriate dietary patterns than with isolated nutrients as broadly applicable immunological interventions. These observations derive predominantly from observational associations, are subject to residual confounding, and should not be interpreted as evidence of causal or disease-preventive effects. The available evidence is insufficient to translate the mapped associations into disease-specific preventive prescriptions, and it does not permit conclusions about the durability of immune effects beyond the follow-up periods examined in the included studies.

These findings may contribute to a hypothesis-generating framework of life-course nutritional immunology, in which childhood diet may interact with microbial, metabolic, genetic, and immune processes over time. Future research should therefore move beyond the search for individual “protective” nutrients and determine whether, and in which dietary environments, developmental windows, and biological contexts, childhood diet is associated with durable immune tolerance and metabolic homeostasis.

## 5. Conclusions

Childhood nutrition is a potential developmental exposure relevant to immune and inflammatory health. Across the 403 studies included in this scoping review, evidence on other immunological outcomes and on middle childhood and adolescence was more limited, and direct mechanistic studies were considerably less common than longitudinal clinical diet–outcome associations.

The findings were most compatible with a context-dependent model in which associations between dietary exposure and immune outcomes vary according to developmental timing, host characteristics, immune state, and the wider dietary and metabolic environment. Multiple interacting pathways were identified, including immune tolerance and sensitization, lipid and micronutrient metabolism, cytokine signaling, the gut microbiome and metabolome, and human-milk-derived bioactive factors.

The diet–microbiome–immune interface provides an important framework for integrating these findings, but few longitudinal human studies have simultaneously measured dietary exposure, microbial or metabolic changes, immune or inflammatory mediators, and later clinical outcomes. Mechanistic interpretation therefore rests mainly on convergence across complementary studies rather than on direct demonstration of complete causal pathways within individual cohorts, and the proposed sequence should be regarded as hypothesis-generating.

Accordingly, the review distinguishes findings directly measured within individual studies, mechanistic interpretations inferred from convergence across studies, and observational diet–outcome associations; none of these establishes a causal immunometabolic programming effect of childhood diet.

Future research should integrate repeated dietary assessments with microbiome and metabolomic profiling, immune phenotyping, inflammatory and metabolic biomarkers, genetic and sex-specific analyses, and standardized long-term clinical outcomes. Greater attention is also needed to dietary exposures during middle childhood and adolescence and to outcomes beyond allergic and atopic disease.

Overall, the evidence is compatible with a life-course nutritional immunology framework in which childhood diet interacts dynamically with microbial, metabolic, genetic, and immune processes over time. This framework is supported mainly for early-life exposures and allergic and atopic outcomes; its extension to other immune-mediated outcomes, to later childhood and adolescence, and to long-term immune programming into adulthood remains to be tested in longitudinal studies. Furthermore, it may help guide future mechanistic and preventive research while avoiding causal or disease-specific dietary conclusions that are not yet supported by longitudinal evidence.

## Figures and Tables

**Figure 1:**
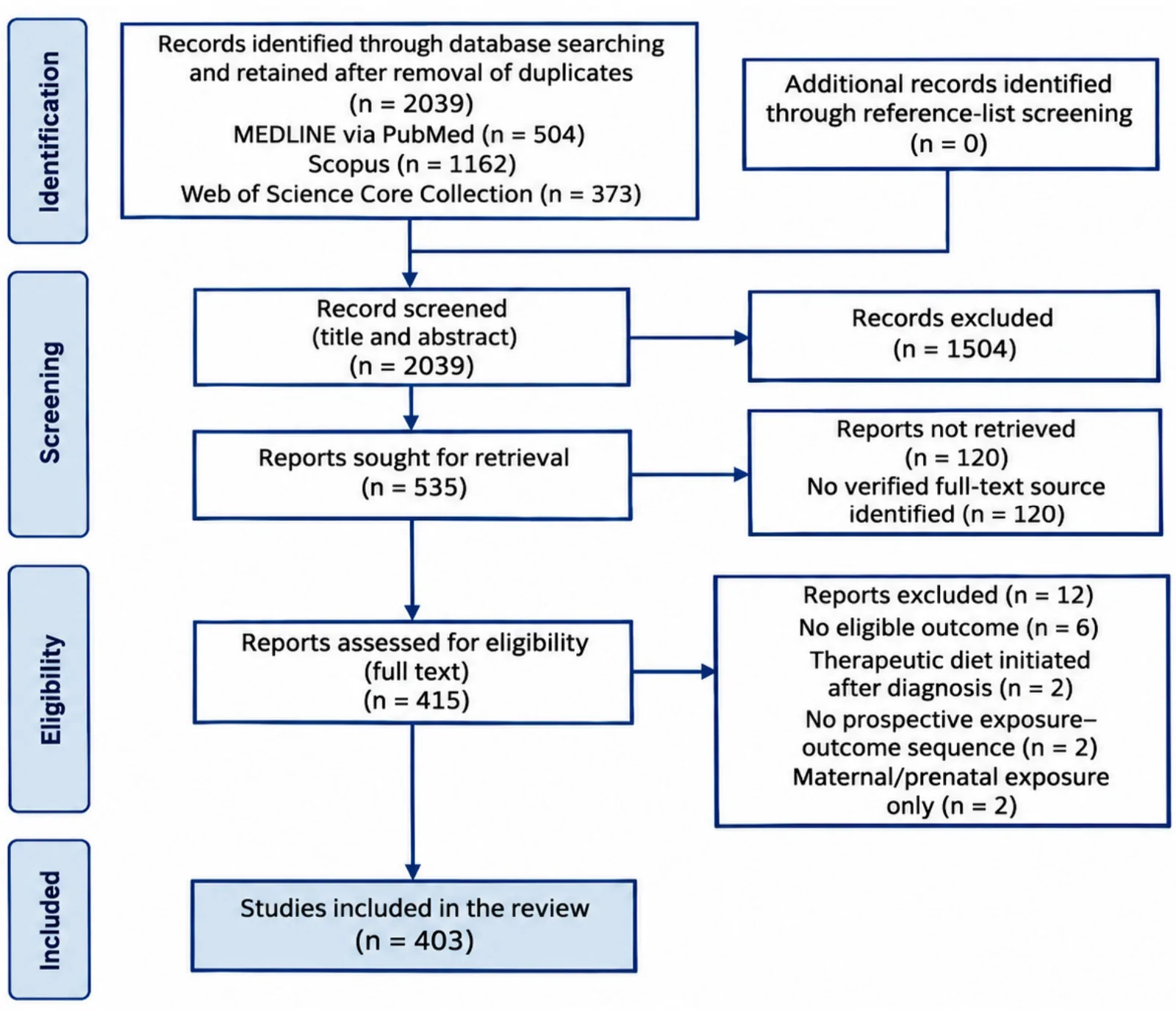
PRISMA-ScR flow diagram of study identification, screening, eligibility assessment, and inclusion.

**Table 1:** Distribution of the included studies (*N* = 403) by study design, region, developmental exposure window, follow-up duration, and primary outcome category, overall and by evidence tier.

Characteristic	Category	All Studies, *n* (%)	Direct (*n* = 30)	Indirect (*n* = 149)	Predominantly Clinical (*n* = 224)
Study design	Prospective/birth cohort	236 (58.6)	22	81	133
	Randomized trial (incl. long-term follow-up)	87 (21.6)	8	43	36
	Case–control (incl. nested)	32 (7.9)	0	7	25
	Retrospective cohort	13 (3.2)	0	8	5
	Non-randomized intervention	5 (1.2)	0	3	2
	Cross-sectional with retrospective exposure data	10 (2.5)	0	5	5
	Other longitudinal design	20 (5.0)	0	2	18
Region	Europe	211 (52.4)	15	83	113
	Asia	75 (18.6)	6	24	45
	North America	51 (12.7)	4	15	32
	Oceania	31 (7.7)	1	17	13
	South America	10 (2.5)	0	3	7
	Africa	4 (1.0)	2	1	1
	Multiple countries	7 (1.7)	0	3	4
	Not reported	14 (3.5)	2	3	9
Developmental exposure window	0–2 years	368 (91.3)	20	131	217
	2–5 years	11 (2.7)	1	9	1
	6–12 years	13 (3.2)	6	5	2
	13–18 years	1 (0.2)	0	0	1
	Repeated/multiple windows	8 (2.0)	1	4	3
	Not reported	2 (0.5)	2	0	0
Follow-up duration	<5 years	173 (42.9)	15	70	88
	5–10 years	101 (25.1)	7	37	57
	>10 years	30 (7.4)	4	13	13
	Outcome assessed in adulthood	14 (3.5)	1	3	10
	Not determined	85 (21.1)	3	26	56
Primary outcome category	Allergic and atopic	398 (98.8)	29	146	223
	Inflammatory and immunological biomarkers	1 (0.2)	0	1	0
	Gastrointestinal and gut-mediated	2 (0.5)	1	1	0
	Immunometabolic (growth)	1 (0.2)	0	0	1
	Other	1 (0.2)	0	1	0

**Table 2:** General characteristics of the studies included in the scoping review.

Characteristic	Evidence Base
Total included studies	403
Publication period	2000–2025
Age at dietary exposure	Birth to 18 years
Predominant developmental period	Infancy and early childhood (0–2 years: 368 of 403 studies; 91.3%)
Main study designs	Birth cohorts, prospective longitudinal cohorts, randomized dietary interventions, nested case–control studies, and other longitudinal observational designs
Main dietary exposure domains	Breastfeeding and human milk; infant formula; complementary feeding; timing of allergenic-food introduction; individual foods and nutrients; fatty acids; vitamins and micronutrients; probiotics/prebiotics; dietary diversity; diet quality; Mediterranean- and Western-type patterns
Main clinical outcome domains	Asthma/wheeze; eczema/atopic dermatitis; food allergy; allergic rhinitis; allergic sensitization
Main mechanistic measurements	IgE/IgA responses; cytokines; immune-cell phenotypes; fatty-acid profiles; human-milk bioactives; gut microbiome; microbial/systemic metabolites; markers of immune tolerance
Direct immunometabolic evidence	30 studies
Mechanistically indirect immunometabolic evidence	149 studies
Predominantly clinical diet–outcome associations	224 studies
Principal mechanistic evidence base	179 studies
Synthesis approach	Descriptive evidence mapping and narrative synthesis

Note: The evidence tiers (direct, indirect, and predominantly clinical) are mutually exclusive and sum to 403 unique studies. Dietary exposures, outcomes, and mechanistic pathways were not mutually exclusive; individual studies could contribute to more than one category, and counts within these categories should not be summed. Study-level characteristics of the 403 included studies are provided in Table S3, and the evidence tier and mechanistic axes of each study are provided in Table S2.

**Table 3:** Distribution of the included studies by follow-up duration and evidence tier.

Follow-Up Duration	Direct (*n* = 30)	Indirect (*n* = 149)	Predominantly Clinical (*n* = 224)	All Studies (*n* = 403), *n* (%)
<5 years	15	70	88	173 (42.9)
5–10 years	7	37	57	101 (25.1)
>10 years	4	13	13	30 (7.4)
Outcome assessed in adulthood	1	3	10	14 (3.5)
Not determined	3	26	56	85 (21.1)

**Table 4:** Diet quality, dietary diversity, and dietary-pattern evidence.

Dietary Construct	Studies, *n* *	Main Outcomes	Direction of Association
Dietary diversity in infancy	8	Food allergy, sensitization, eczema; gut microbial richness	Predominantly inverse (favorable); not uniform
Dietary patterns (empirical or predefined)	7	Asthma/wheeze, rhinitis, food allergy	Heterogeneous
Composite diet-quality scores	3	Sensitization, allergic disease, food allergy	Mixed
Mediterranean-type pattern	1	Asthma, recurrent childhood outcomes	Possibly favorable; limited evidence
Antioxidant-rich diet/TAC	2 †	IgE, sensitization, allergic asthma	Generally favorable
Fruit- and vegetable-based pattern	1	Sensitization, IgE	Favorable
Western-type pattern	‡	Wheeze, respiratory symptoms	Possibly unfavorable; partly explained by energy intake
Ultra-processed food (early life)	1	Respiratory morbidity	No independent association

* Number of studies contributing to each construct. Constructs overlap, and a study may contribute to more than one row; counts are therefore not additive and exceed the 17 unique studies in this domain. † One plant-based/antioxidant study from the diet-quality mapping and one mechanistic study of total dietary antioxidant capacity. ‡ Assessed within the dietary-pattern studies; not counted separately. The immunometabolic relevance of each construct is discussed in Section 4.9. Abbreviations: IgE, immunoglobulin E; TAC, total antioxidant capacity.

**Table 5:** Immunometabolic evidence map across the six principal mechanistic axes.

Mechanistic Axis	Studies, *n* *	Direct/Indirect/Predominantly Clinical †	Principal Dietary Exposures	Main Mechanisms/Biomarkers	Main Related Outcomes	Overall Pattern
Allergic sensitization, IgE/IgA responses, and immune tolerance	156	25/120/11	Breastfeeding, formula, allergen introduction, complementary feeding, specific foods, probiotics/prebiotics	IgE, IgA, skin-prick sensitization, allergen-specific responses, IgG4, tolerance-related measures	Food allergy, eczema/AD, asthma/wheeze, rhinitis, sensitization	Associations varied according to exposure timing, immune state, microbial environment, and feeding context
Lipid immunometabolism and fatty-acid pathways	20	11/9/0	Human-milk fatty acids, n-3/n-6 PUFA, EPA/DHA, fish, formula lipids	Fatty-acid metabolism, FADS pathways, DPP10 interactions, inflammatory lipid signaling, ILC3 activation	Asthma, atopy, eczema, rhinitis, sensitization	Heterogeneous and modified by pathway, genotype, and context
Vitamins, micronutrients, and antioxidant metabolism	15	11/4/0	Vitamins A and D, beta-carotene, antioxidant-rich foods, total antioxidant capacity	Redox balance, vitamin-dependent immune signaling, IgE modulation, inflammatory regulation	Sensitization, asthma, atopy, allergic disease	Whole-diet antioxidant exposure showed a more consistent favorable direction than isolated supplementation
Cytokine signaling and immune-cell programming	13	2/11/0	Breastfeeding/human milk, allergenic-food exposure, fish-oil supplementation, micronutrients	TGF-β, IL-10, IL-1β, TNF-α, IL-2, IL-5, sCD14, T-cell-related measures	Eczema, sensitization, atopy, immune maturation	Early dietary exposures were associated with differences in inflammatory and regulatory immune signaling
Gut microbiome, metabolomics, and microbial metabolites	14	8/4/2	Breastfeeding, formula, dietary diversity, probiotics, prebiotics	Bifidobacteria, microbial richness, microbial-derived metabolites, amino-acid metabolism, systemic metabolomics	Food allergy, IgE sensitization, asthma, eczema	Multiple studies linked dietary exposure with microbial/metabolic changes and subsequent immune phenotypes
HMOs and other milk-derived bioactive signals	10 228	3/6/1	Human milk, HMOs, prebiotic oligosaccharide-supplemented formula	2′-FL, 6′-SL, IgA, lactoferrin, TGF-β, bifidogenic effects, mucosal signaling	Eczema, food allergy, sensitization, immune maturation	Associations differed according to individual components and feeding context
Total axis assignments †	228	60/154/14	Unique studies in the review: 403 (30 direct/149 indirect/224 predominantly clinical); axis assignments exceed unique studies because axes overlap			

* Number of unique studies mapped to each axis. Mechanistic axes overlap: a study could be mapped to more than one axis and was counted once within each axis to which it was mapped; counts should therefore not be summed. The axes assigned to each study are listed in Table S2. † Evidence tiers are mutually exclusive (Section 2.9): direct, a metabolic or nutritional pathway and an immune or inflammatory component measured in the same study; indirect, an intermediate biological pathway measured on only one side of the immunometabolic interface; predominantly clinical, no defined intermediate mediator measured. Within each axis, the three-tier counts sum to the axis total; the totals row gives the sum of axis assignments (228). Abbreviations: AD, atopic dermatitis; DHA, docosahexaenoic acid; DPP10, dipeptidyl peptidase-like 10; EPA, eicosapentaenoic acid; FADS, fatty acid desaturase; FL, fucosyllactose; HMO, human-milk oligosaccharide; Ig, immunoglobulin; IL, interleukin; ILC3, group 3 innate lymphoid cells; PUFA, polyunsaturated fatty acid; sCD14, soluble CD14; SL, sialyllactose; TGF-β, transforming growth factor beta; TNF-α, tumor necrosis factor alpha.

**Table 6:** Level of evidence supporting each step of the proposed diet–immune pathway.

Step of the Proposed Pathway	Directly Observed Within Included Studies (Examples)	Not Directly Demonstrated/Proposed on The Basis of Convergence Across Studies or External Literature	Interpretation Level
Diet → gut microbiota composition	Breastfeeding was associated with early microbial colonization, including enrichment of bifidobacterial taxa; in one cohort, enrichment of B. longum subsp. infantis was associated with attenuation of antibiotic-related microbiome disruption and with subsequent childhood asthma risk [24]. Lower infant dietary diversity was associated with lower gut microbial richness and subsequent food-allergy development [27].	Relative contribution of specific dietary components, as distinct from concurrent factors (e.g., antibiotic use, delivery mode), is not resolved. Prebiotic/probiotic interventions changed composition or function in several studies, but overall community structure was not consistently altered.	Directly measured (diet–microbiota); immune mediation = plausible mechanistic interpretation
Diet → microbial and systemic metabolites	Urinary metabolites, including microbial-derived and amino-acid-related compounds, were associated with formula exposure and with subsequent milk sensitization [25].	Direct measurements of individual short-chain fatty acids were not sufficiently consistent to support an SCFA-specific category. The role of SCFAs in immune regulation and regulatory T-cell development is supported by external mechanistic literature [36,37,38], not by the included studies.	Directly measured for selected metabolites; SCFA-mediated immune regulation = plausible mechanistic interpretation (external literature)
Diet/human milk → cytokine and immune-cell signaling	Breastfed infants showed a more anti-inflammatory cytokine profile than formula-fed infants (lower circulating TNF-α and IL-2, higher TGF-β) [22]; milk-derived factors were related to subsequent eczema, atopy, or immune maturation [21]; fish-oil supplementation modified associations between traffic-related air pollution, allergen sensitization, and allergen-specific IL-5 responses [23].	Mediation of later clinical outcomes by these differences in immune signaling is not established by the studies summarized.	Directly measured (diet–immune markers); link to later clinical outcome = plausible mechanistic interpretation
Dietary antigen exposure → tolerance or sensitization	In a trial of early egg introduction, repeated exposure was associated with tolerance-related changes, including increased allergen-specific IgG4, while a substantial proportion of high-risk infants were already sensitized before or at first exposure [12].	The direction of effect appears to depend on the immune state at the start of exposure; generalization to other allergens, populations, or feeding contexts is not established.	Directly measured in the allergen and population studied (randomized trial); generalization = plausible mechanistic interpretation
Lipids and fatty acids → immune signaling	Long-chain saturated fatty acids in human milk were associated with inflammatory ILC3-related responses [15]. Gene–diet interactions were reported for EPA/DHA intake and FADS genotype with incident asthma [13], and for omega-3 exposure and DPP10-region variants with childhood atopy [14].	Mechanisms underlying effect modification were not demonstrated; null findings were common in observational studies and trials, and milk PUFA composition did not consistently account for the effects of breastfeeding.	Observational association/interaction; mechanism = plausible mechanistic interpretation
Intestinal barrier	Intestinal-barrier markers were charted where available, but no barrier-specific findings are synthesized in the present review.	The barrier step is included in the conceptual pathway on the basis of background literature [1,2,3].	Plausible mechanistic interpretation only; not addressed by the findings synthesized
Complete sequence (diet → microbiota/metabolites → immune programming → clinical phenotype) within the same cohort	Relatively few individual studies measured the complete sequence within the same population; most assessed selected components (e.g., diet and clinical allergy, diet and microbiome, diet and immune biomarkers).	The mechanistic evidence arises predominantly from convergence across complementary studies. Only 30 of 403 studies (7.4%) provided relatively direct immunometabolic evidence.	Plausible mechanistic interpretation (convergence across studies)

Note: The arrows (→) indicate the proposed direction of relationships between successive components of the conceptual pathway and do not necessarily imply causality. Directly observed associations and hypothesized mechanistic links are distinguished in the corresponding columns. Reference numbers correspond to the reference list of the manuscript. SCFA, short-chain fatty acid; EPA, eicosapentaenoic acid; DHA, docosahexaenoic acid; FADS, fatty acid desaturase; ILC3, group 3 innate lymphoid cell; PUFA, polyunsaturated fatty acid.

## Data Availability

The data supporting the findings of this scoping review, including the complete search strategies and the record-level screening, selection, and classification data, are available within the article and its Supplementary Materials (Tables S1–S6 and File S1).

## Supplementary Materials

The following supporting information can be downloaded at: https://www.mdpi.com/article/10.3390/nu18213706/s1; Table S1: Database-specific search strategies; Table S2: Classification of each included study (*n* = 403) by evidence tier and mechanistic axis; Table S3: Characteristics of the included studies (*n* = 403); Table S4: Reports sought for retrieval but not retrieved (*n* = 120) and reasons for non-retrieval; Table S5: Reports excluded at full-text assessment (*n* = 12) and primary reasons for exclusion; Table S6: All records screened (*n* = 2039), with screening decision, selection stage, and reason for exclusion.
