# Supplementary material for: From Early Diet to Immune Phenotype: Childhood Nutrition and Immunometabolic Programming Across the Life Course—A Scoping Review
Source: Nutrients. 2026 Nov 8;18(21):3706. doi: 10.3390/nu18213706 (PMC13765458; doi:10.3390/nu18213706)
Supplement: Supplementary file 1 [file nutrients-18-03706-s001.zip › Supplementary Table S1.pdf]

Table S1. Database-specific search strategies

Database (platform)	Search string	Limits
MEDLINE (PubMed)	#1 ("Infant"[Mesh] OR "Child"[Mesh] OR "Adolescent"[Mesh] OR infan*[tiab] OR newborn*[tiab] OR neonat*[tiab] OR toddler*[tiab] OR preschool*[tiab] OR child*[tiab] OR adolescen*[tiab] OR "early life"[tiab])<br>#2 ("Breast Feeding"[Mesh] OR "Infant Formula"[Mesh] OR "Infant Nutritional Physiological Phenomena"[Mesh] OR "Diet"[Mesh] OR "Feeding Behavior"[Mesh] OR "Dietary Supplements"[Mesh] OR breastfe*[tiab] OR "breast milk"[tiab] OR "human milk"[tiab] OR "infant formula"[tiab] OR "complementary feeding"[tiab] OR weaning[tiab] OR "solid food*"[tiab] OR "food introduction"[tiab] OR diet*[tiab] OR nutrition*[tiab] OR "dietary pattern*"[tiab] OR "diet quality"[tiab] OR "dietary diversity"[tiab] OR "fatty acid*"[tiab] OR vitamin*[tiab] OR probiotic*[tiab] OR prebiotic*[tiab] OR "ultra-processed"[tiab])<br>#3 ("Hypersensitivity"[Mesh] OR "Asthma"[Mesh] OR "Dermatitis, Atopic"[Mesh] OR "Food Hypersensitivity"[Mesh] OR "Rhinitis, Allergic"[Mesh] OR "Immunoglobulin E"[Mesh] OR "Autoimmune Diseases"[Mesh] OR "Inflammatory Bowel Diseases"[Mesh] OR "Celiac Disease"[Mesh] OR "Inflammation"[Mesh] OR "Cytokines"[Mesh] OR "Gastrointestinal Microbiome"[Mesh] OR "Non-alcoholic Fatty Liver Disease"[Mesh] OR allerg*[tiab] OR atop*[tiab] OR asthma*[tiab] OR wheez*[tiab] OR eczema*[tiab] OR "atopic dermatitis"[tiab] OR sensiti*ation[tiab] OR IgE[tiab] OR autoimmun*[tiab] OR inflammat*[tiab] OR cytokine*[tiab] OR "immune programming"[tiab] OR immunometabol*[tiab] OR microbio*[tiab] OR "fatty liver"[tiab] OR cognit*[tiab])<br>#4 #1 AND #2 AND #3	English; 2000/01/01–2025/12/31; Humans
Scopus (Elsevier)	TITLE-ABS-KEY(infan* OR newborn* OR neonat* OR toddler* OR preschool* OR child* OR adolescen* OR "early life") AND TITLE-ABS-KEY(breastfe* OR "breast milk" OR "human milk" OR "infant formula" OR "complementary feeding" OR weaning OR "solid food*" OR "food introduction" OR diet* OR nutrition* OR "dietary pattern*" OR "diet quality" OR "dietary diversity" OR "fatty acid*" OR vitamin* OR probiotic* OR prebiotic* OR "ultra-processed") AND TITLE-ABS-KEY(allerg* OR atop* OR asthma* OR wheez* OR eczema* OR "atopic dermatitis" OR sensiti*ation OR ige OR autoimmun* OR inflammat* OR cytokine* OR "immune programming" OR immunometabol* OR microbio* OR "fatty liver" OR cognit*)	LANGUAGE(English); PUBYEAR > 1999 AND PUBYEAR < 2026; DOCTYPE(ar)
Web of Science Core Collection (Clarivate)	TS=(infan* OR newborn* OR neonat* OR toddler* OR preschool* OR child* OR adolescen* OR "early life") AND TS=(breastfe* OR "breast milk" OR "human milk" OR "infant formula" OR "complementary feeding" OR weaning OR "solid food*" OR "food introduction" OR diet* OR nutrition* OR "dietary pattern*" OR "diet quality" OR "dietary diversity" OR "fatty acid*" OR vitamin* OR probiotic* OR prebiotic* OR "ultra-processed") AND TS=(allerg* OR atop* OR asthma* OR wheez* OR eczema* OR "atopic dermatitis" OR sensiti*ation OR IgE OR autoimmun* OR inflammat* OR cytokine* OR "immune programming" OR immunometabol* OR microbio* OR "fatty liver" OR cognit*)	Language: English; publication years 2000–2025; document type: article

Note for the supplementary file: “The search was reported according to PRISMA-S (Rethlefsen, M.L.; Kirtley, S.; Waffenschmidt, S.; et al. Syst. Rev. 2021, 10, 39). Records from the three databases were merged and deduplicated sequentially by DOI, exact normalized title, and high-similarity title matching with manual audit, yielding 2,039 unique records (PubMed, n = 504; Scopus, n = 1,162; Web of Science, n = 373). Reference lists of included studies and relevant reviews were screened; no additional records were identified.”
